# Non-volatile conductive gels made from deep eutectic solvents and oxidised cellulose nanofibrils†

**DOI:** 10.1039/d0na00976h

**Published:** 2021-03-02

**Authors:** Saffron J. Bryant, Marcelo A. da Silva, Kazi M. Zakir Hossain, Vincenzo Calabrese, Janet L. Scott, Karen J. Edler

**Affiliations:** Department of Chemistry, University of Bath Claverton Down Bath BA2 7AY UK saffron.bryant@rmit.edu.au k.edler@bath.ac.uk; Centre for Sustainable Chemical Technologies, University of Bath Claverton Down Bath BA2 7AY UK; School of Science, College of Science, Engineering and Health, RMIT University Melbourne VIC 3001 Australia

## Abstract

Ionogels offer huge potential for a number of applications including wearable electronics and soft sensors. However, their synthesis has been limited and often relies on non-renewable or non-biocompatible components. Here we present a novel two-component ionogel made using just deep eutectic solvents (DESs) and cellulose. DESs offer a non-volatile alternative to hydrogels with highly tuneable properties including conductivity and solvation of compounds with widely varying hydrophobicity. DESs can be easily made from cheap, biodegradable and biocompatible components. This research presents the characterisation of a series of soft conductive gels made from deep eutectic solvents (DESs), specifically choline chloride-urea and choline chloride-glycerol, with the sole addition of TEMPO-oxidised cellulose nanofibrils (OCNF). A more liquid-like rather than gel-like conductive material could be made by using the DES betaine–glycerol. OCNF are prepared from sustainable sources, and are non-toxic, and mild on the skin, forming gels without the need for surfactants or other gelling agents. These DES-OCNF gels are shear thinning with conductivities up to 1.7 mS cm^−1^ at ∼26 °C. Given the thousands of possible DESs, this system offers unmatched tunability and customisation for properties such as viscosity, conductivity, and yield behaviour.

## Abstract 2

Conductive, shear-thinning gels made from deep eutectic solvents and cellulose nanofibrils.

## Graphical abstract

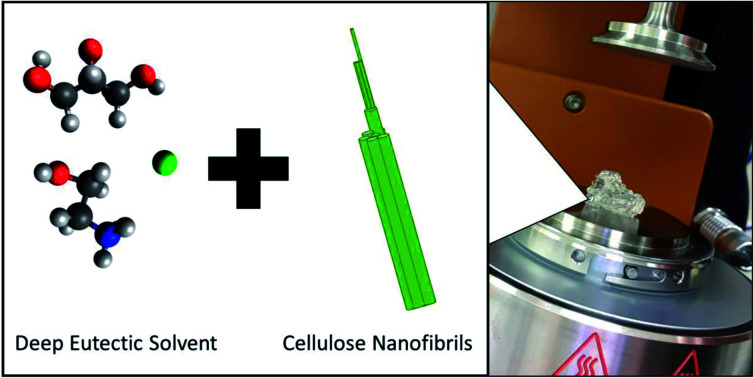

## Introduction

Conductive gels offer promising applications *e.g.* as soft sensors, in energy storage, and wearable electronics.^1–5^ Hydrogels with ionic species have some potential in this area. However, they suffer from dehydration due to evaporation when exposed to the air which limits their applications and lifespan.^3^

Ionogels offer a solution to the dehydration issue by using non-volatile ionic liquids instead of water as dispersing media.^6–8^ Ionic liquids are molten salts that are liquid at room temperature. They have been combined with polymers such as poly(vinyl alcohol),^9^ poly(ethylene glycol) diacrylate^7,10^ and polysaccharides such as chitin, guar gum and agarose to create conductive gels.^6,11^ The properties of these gels vary depending on the polymer and ionic liquid used, which allows tuning and tailoring of the gels to the intended applications. However, there are two main issues with ionic liquid based ionogels that prevent wider application. Firstly, many of the synthesis methods may require energetically costly processes, *e.g.* heating at 100 °C for 10 hours, and the ionic liquids themselves can be costly and time-consuming to make.^11^ Secondly, ionic liquids, and their preparation, can be toxic or generate toxic waste,^12^ so ionic liquids must be carefully chosen when seeking ionogels for potential applications in healthcare, personal/home care or foodstuffs.

Deep eutectic solvents (DESs) are a subclass of neoteric liquids, like ionic liquids, which are made of a hydrogen bond donor and a hydrogen bond acceptor.^13^ This subclass are often easier to make than many ionic liquids (simple mixing of components in mild conditions), and can be made of cheap and widely available components, such as food additives, with no waste products from synthesis due to 100% atom utilisation.^14,15^ Thus, deep eutectic solvents can be cheap, green, non-toxic and biodegradable.^16,17^ Deep eutectic solvents have a hydrogen bonded nanostructure that persists, even after addition of water.^18,19^

Due to its novelty, research on conductive gels using deep eutectic solvents (aka eutectogels) is limited. Some works have used deep eutectic solvents as plasticisers for starch or cellulose to create conductive films with improved mechanical properties.^20–22^ Others have used DESs as polymerisation media, *e.g.* of 2-hydroxyethyl methacrylate^23,24^ or poly(ethylene glycol) diacrylate,^25^ to obtain eutectogels. Eutectogels have also been made using amino acids and choline-chloride/phenylacetic acid-based DESs.^26^ A very promising eutectogel was made with choline chloride-ethylene glycol and gelatin, which had high conductivity and good mechanical properties.^3^ However, this method required heating to 70 °C and subsequent refrigeration. In order to be more environmentally friendly, we are interested in making gels using plant-based materials, rather than gelatin which is sourced from animal tissue and therefore may raise religious or ethical concerns, and using processes that rely on mild conditions, giving potential for future incorporation of sensitive molecular species.

**Fig. 1:**
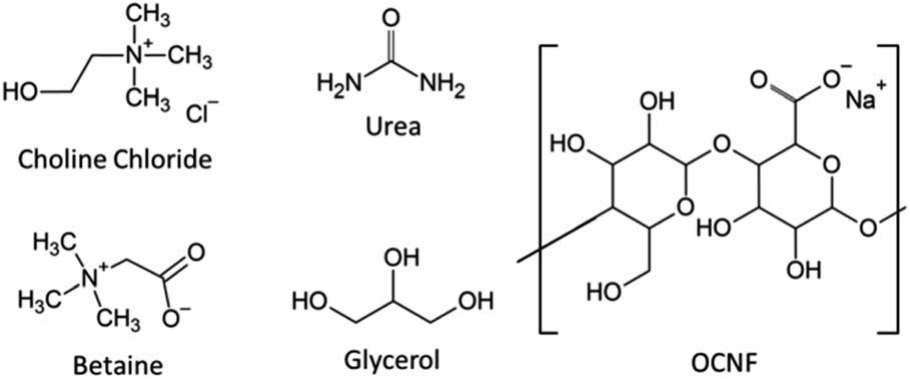
Chemical structures of the DES components and partially oxidised cellulose nanofibrils (OCNF) used in this work.

Partially oxidised cellulose nanofibrils (OCNF) (Fig. 1) produced *via* TEMPO-mediated oxidation have a large aspect ratio (5–10 nm cross-section and 100 nm to several μm in length). A high negative surface charge (−60 mV in *ζ* potential), due to the carboxyl groups on the fibril surface, makes it easy to obtain stable dispersions of these fibrils in water.^27,28^ These fibrils and related products have been extensively characterised in previous studies and so are not analysed further here.^27–31^ Previous work has demonstrated rheological modification and gel formation using OCNF by our group and many others in aqueous systems, including in the presence of co-solutes (salt and surfactants) and co-solvents (alcohols) or with changes in the solvent (pH and temperature).^27,28,32–39^ The biocompatibility of OCNF was recently reviewed and it was found to have low toxicity even in oral application.^40^ Gels utilising OCNF have shown successful drug penetration across pig skin,^41^ and OCNF particles have been studied as potential drug carriers.^42^

The work presented here examines the formation of eutectogels using OCNF, exploring the use of OCNF as a rheological modifier in non-aqueous media. The effect of changing either the hydrogen bond donor (choline chloride or betaine) or the hydrogen bond acceptor (glycerol or urea) of the DES on gel properties is explored, along with the effect of temperature. Temperature is an important factor to consider as different applications may require different temperatures, and the effect of changing temperature on gel properties should be understood.

The three DESs chosen provide adequate comparison of the effects of changing either the hydrogen bond donor or the acceptor for these preliminary results. Future work will expand to a larger library of DESs to even better elucidate the effect of each DES component.^17^ Furthermore, the chosen DES components are generally considered harmless as per the safety data sheets and several toxicity studies.^17^ However, as with any chemical, cytotoxicity varies significantly depending on concentration, cells tested against, and the specific combination of components and this would have to be taken into consideration for any clinical applications.^16,17,43–45^

We found good gel formation with tuneable properties and straightforward synthesis.

## Experimental

TEMPO-mediated oxidation was used to produce partially oxidised cellulose nanofibrils as previously described.^27,28^ These were obtained as in our previous work as a *ca.* 8 wt% solids paste in water.^33,35^ The degree of oxidation of these fibrils is 25% (number of carboxylate groups compared to total anhydroglucose units), as determined by conductometric titration.^46^ The fibrils are 4–12 nm in diameter (reflecting their elliptical cross section) and with an average length of 160 ± 60 nm, as measured by TEM.^33^

Dialysis against deionised water (18.2 MΩ cm) removed residual salts and preservatives from OCNF. The OCNF was then freeze-dried and resuspended to 1.5 wt% in deionised water before dispersal by sonication (Ultrasonic Processor, FB-505, Fisher – 550 W), at 30% amplitude with 1 s on 1 s off pulses, for ∼1 hour or until the dispersion became transparent.

Three deep eutectic solvents were used. Choline chloride–urea (ChCl–U) was synthesised by combining choline chloride (Sigma, ≥98%) and urea (Sigma, ≥99.5%) at a 1 : 2 mol ratio. Betaine–glycerol (betaine–Gly) was synthesised by combining betaine (Sigma, ≥99%) and glycerol (Sigma, ≥99%) in a 1 : 2 mol ratio. Choline chloride–glycerol (ChCl–Gly) was synthesised by combining choline chloride and glycerol in a 1 : 2 mol ratio. All these DESs were stirred at 50 °C to form a homogenous liquid and then placed in a freeze-dryer to remove any residual water.

Addition of dried OCNF directly to the DESs was not possible as the strong gelation made subsequent uniform dispersion of the fibrils by simple mixing unfeasible. Therefore, each DES was combined with an equal weight of 1.5 wt% OCNF in water, mixed thoroughly, and then freeze-dried to remove the water. In all cases the water content of the final gel was ≤2 wt%.

Rheological measurements of each sample were conducted using a stress-controlled Discovery Hybrid Rheometer, Model HR-3 (TA Instruments) equipped with a sand-blasted 40 mm parallel plate geometry. Measurements were made at 25, 45 and 65 °C using a Peltier unit (±0.1 °C). Measurements were made as follows: frequency sweeps were performed at 0.1% strain covering an angular frequency from 0.01 to 50 rad s^−1^, amplitude sweeps were performed at a fixed angular frequency of 6.28 rad s^−1^ across a strain range from 0.01 to either 100% or 1000%. Flow sweeps were performed at shear rates from 0.01 to 1 s^−1^ due to the early onset of edge failure and normal force effects, flow sweeps could not be measured at shear rates higher than 1 s^−1^. The DESs on their own are Newtonian fluids and were not further investigated.

SAXS measurements were performed on an Anton-Paar SAXSPoint 2.0 provided by the Material and Chemical Characterisation Facility (MC^2^)^47^ equipped with a copper source (Cu Kα, *λ* = 1.542 Å) and a 2D EIGER R series Hybrid Photon Counting (HPC) detector. The sample-detector distance was 556.9 mm covering *q* range of about 0.008–0.4 Å^−1^. The DESs on their own were loaded into 1 mm quartz capillaries, while the DES–OCNF mixtures, because of their gel-like nature, were loaded into paste cells with polycarbonate windows. Data was collected in one frame, with 900 s exposure, then processed. Scattering from an empty cell was used for background subtraction. Fitting was performed using SASView (version 4.2.1, see http://www.sasview.org/for more information) (models detailed in ESI†). Measurements of the DES–OCNF mixtures were made at 25, 45, and 65 °C using a Peltier unit (±0.1 °C) for temperature control.

Conductivity measurements were made using a Mettler Toledo SevenMulti™. The mixtures were gradually heated from room temperature (∼26 °C) to ∼65 °C and conductivity measurements taken at regular intervals. Measurements were taken in triplicate for each temperature.

## Results and discussion

### Rheology

All three of the chosen DESs, choline chloride–urea (ChCl–U), choline chloride–glycerol (ChCl–Gly), and betaine–glycerol (betaine–Gly), are viscous liquids on their own, but addition of OCNF to any of them resulted in immediate thickening. In the case of ChCl–Gly and ChCl–U, self-standing physical gels formed with OCNF after water removal by freeze-drying. For betaine–Gly with OCNF a sticky and highly viscous liquid formed after freeze-drying.

Addition of salt to dispersions of OCNF in water at such high ionic strengths as are present in these DESs results in aggregation and precipitation of the fibrils.^36^ It is interesting to note that such precipitation does not occur in these DESs, suggesting that interactions beyond simple electrostatic shielding are present.

These visual observations are further supported by the rheological measurements. As shown in Fig. S1–S3,†*G*′ (storage modulus) and *G*′′ (loss modulus) of the mixtures made with OCNF and either ChCl–U or ChCl–Gly had only small frequency dependence and in all cases, *G*′ was significantly higher than *G*′′. In contrast, *G*′ and *G*′′ of the sample made with betaine–Gly had much greater frequency dependence and a *G*′ and *G*′′ cross-over present in the frequency range measured. This cross-over shifted toward lower frequencies with decreasing temperature, indicating an increased relaxation time. A functional definition of a gel, in rheological terms, is where *G*′ and *G*′′ are not frequency dependent over a significant frequency window, and that *G*′ > *G*′′.^48^ Such rheological behaviour is normally observed in chemical, or permanent, gels, where the gel network is maintained by permanent or long-lived connections such as covalent bonds. However, it can also be observed for the so-called physical gels, where the gel network is maintained by transient or short-lived connections. In this case, a small degree of frequency dependence is observed.^49^ Therefore, based on the above rheological measurements it is likely that the mixtures of OCNF with both ChCl–U and ChCl–Gly formed physical gels with transient or short-lived connections, while OCNF mixed with betaine–Gly formed a liquid, albeit a very thick and viscous one.

Tan(*δ*) is the ratio of *G*′′ to *G*′ and is an easy way to compare the ratio of the viscous to elastic behaviour in these systems. It is a convenient value for following changes in gel properties while ignoring frequency-dependent changes. Tan(*δ*) was taken from the frequency sweep curves (Fig. S1–S3†) at an angular frequency of 6.28 rad s^−1^ (equivalent to 1 Hz). Tan(*δ*) values less than 1 are associated with more gel-like behaviour. The lower the value, the more predominant the solid, or elastic, contribution.

**Fig. 2:**
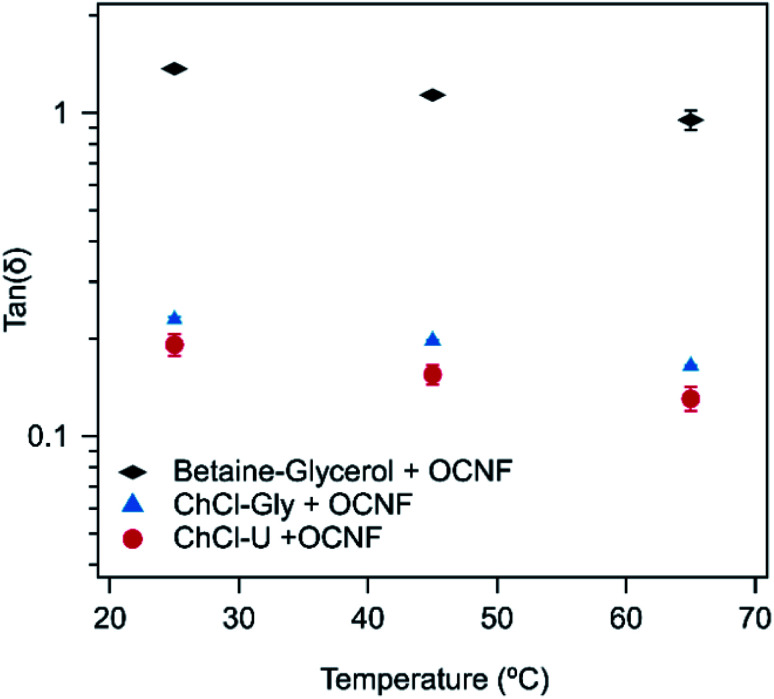
Tan(*δ*) of 1.5 wt% OCNF with either ChCl–U, ChCl–Gly or betaine–Gly at 25, 45, or 65 °C. Error bars are derived from the standard deviation of duplicate measurements.

As shown in Fig. 2, the tan(*δ*) of all three mixtures decreased with increasing temperature. When looking at the individual contributions of *G*′ and *G*′′ in the frequency sweeps, *G*′′ decreased to a greater degree than *G*′ with increasing temperature. This indicates the dissipative processes, responsible for *G*′′, are more easily disrupted by increasing temperature, thus reducing *G*′′, while leaving *G*′ more or less unchanged and increasing overall brittleness of the systems. In typical gels formed from aggregated rods kept together *via* intermolecular interactions, an increase in temperature would be expected to lead to an increase in tan(*δ*), as these interactions would be disrupted by the temperature increase. However, we note that for OCNF in water, an increase in *G*′ occurs upon heating,^32^ causing a transition in tan(*δ*) to values below one (*i.e.* the system gels), so the decrease in tan(*δ*) is not unusual for suspensions of this material. In water this is attributed to the decrease in water dielectric constant upon heating, altering the extent of electrostatic screening in the system, which enables greater aggregation of the fibrils,^32^ allowing gelation, or strengthening gels which already exist. Similar effects may be also important in these OCNF eutectogels since the dielectric constants of ChCl–U and ChCl–Gly have similarly been shown to decrease with increasing temperature.^50^ Other relaxation processes may also be occurring. For example, if configurational entropy^48^ is a relevant contribution to *G*′, it would lead to a tan(*δ*) decrease with increasing temperature such as is observed here, as entropic elasticity increases with temperature. This is a likely explanation, but further certainty regarding gel structure cannot be obtained from rheological measurements alone.

In the frequency sweeps (Fig. S1–S3†) there appears to be a bigger change between 25 and 45 °C than between 45 and 65 °C. It is likely that the changes in gel structure which cause a change in *G*′′ and *G*′ are more significant in the lower temperature range and so further heating causes less obvious changes.

As expected, based on visual observations, the samples made with betaine–Gly had much higher tan(*δ*) values than samples made with ChCl–U or ChCl–Gly. In contrast, samples made with either ChCl–U or ChCl–Gly had very similar tan(*δ*) values at each temperature, with the values for ChCl–U being slightly lower.

In water, 1.5 wt% OCNF at 25 °C forms a viscous liquid with a tan(*δ*) of approximately 1.3 (calculated from data shown in Fig. S4†), which is the same as for the betaine–Gly system presented here, but much higher than that of the ChCl–U and ChCl–Gly systems. This suggests that interactions between OCNF and these two DESs are causing gelation and thickening, but that the same degree of networking is not occurring in the betaine–Gly system.

In water, the tan(*δ*) of OCNF dispersions can be decreased by addition of salt which facilitates closer association and networking of fibrils, leading to increased gelation.^33^ Thus, charge screening is a key mechanism for gelation of OCNF dispersions. The main difference between ChCl–Gly and betaine–Gly is that in choline chloride the charges are physically located in two independent ions, choline and chloride, whilst the betaine molecule carries both charges. In addition, the betaine molecule has a carboxylic group while the choline molecule has a hydroxyl group. Thus, the charge interactions of these two compounds will differ. It is possible that the choline cation is better able to interact with the carboxylate groups of the OCNF fibrils, thus preventing fibril–fibril electrostatic repulsion and resulting in increased gel-like properties as is observed upon addition of salt to aqueous dispersions of OCNF.^33^

Amplitude sweeps are used to examine the linear viscoelastic region and also to determine the yield strain of a system. For this research, the yield strain is defined as the point of strain at which *G*′ and *G*′′ cross-over in the amplitude sweep. These sweeps are presented in the ESI (Fig. S5–S7†).

**Fig. 3:**
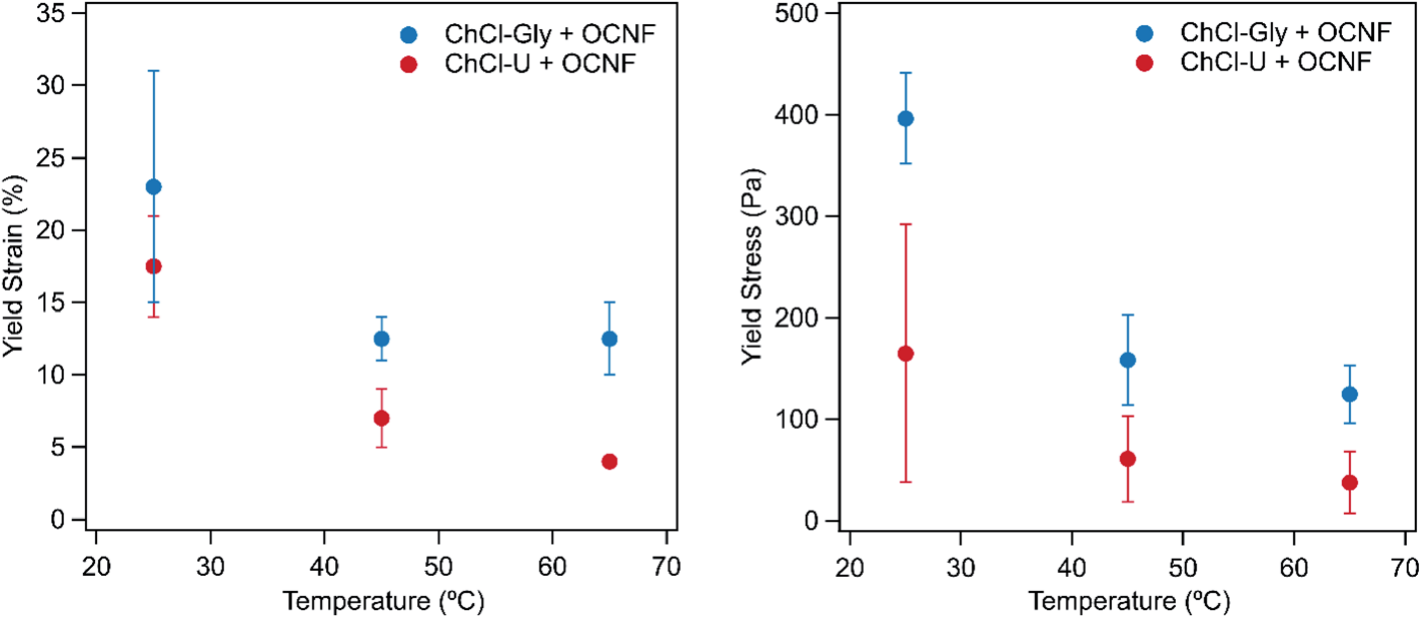
The yield strain (left) and yield stress (right) values of 1.5 wt% OCNF with ChCl–U or ChCl–Gly at 25, 45, or 65 °C. Error bars are based on the standard deviation of duplicate measurements.

Fig. 3 shows the yield strain and yield stress for OCNF mixtures with ChCl–U and ChCl–Gly at different temperatures. The yield values for OCNF with betaine–Gly could not be determined because *G*′ and *G*′′ do not cross within the yield strain region measured, as expected as this mixture did not form a gel. Similarly, 1.5 wt% OCNF in water does not have a measurable yield strain or stress in the region measured. Both the yield strain and yield stress of the ChCl–Gly mixtures are consistently higher than that of ChCl–U mixtures at a given temperature. This suggest that in ChCl–Gly the system is more elastic and stronger than the ChCl–U system.

Both yield stress and yield strain decreased with increasing temperatures for both DES:OCNF mixtures. However, the large errors, due to high batch-to-batch variation and loading, should be noted.

These gels have more favourable rheological properties than that found previously for eutectogels. For example, the yield strain for gels made with ChCl–U and ChCl–Gly are higher, and the tan(*δ*) values of these gels is much lower (<0.2), than that obtained previously for gels made with choline chloride-phenylacetic acid and the l-amino acids isoleucine and tryptophan.^26^ This may be because OCNF is made of long fibrils which are much more able to form a persistent three dimensional network than the single amino-acid residues used in the previous study.

**Fig. 4:**
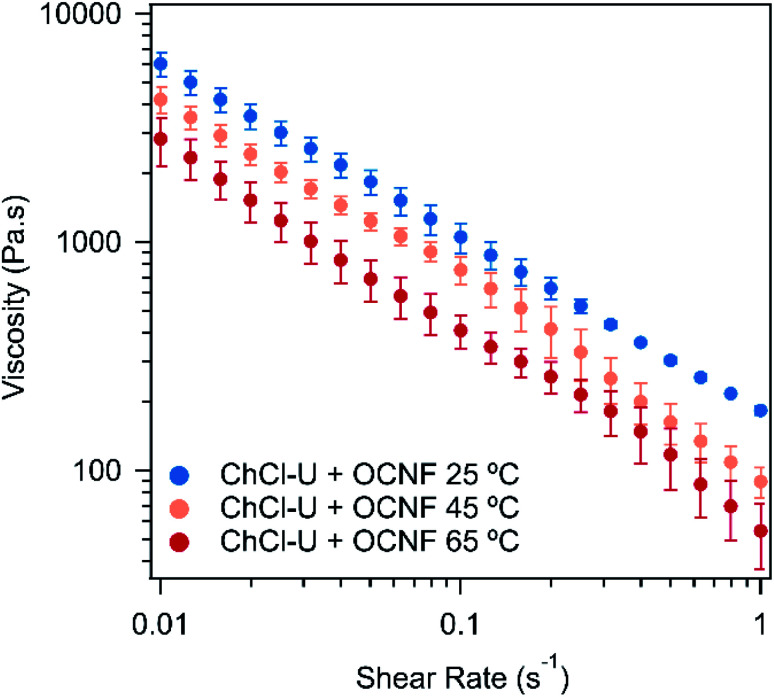
Flow curves of 1.5 wt% OCNF in ChCl–U at 25, 45, or 65 °C. Error bars are based on the standard deviation of duplicate measurements.

One of the most useful rheological measurements for industrial applications is viscosity, especially across a range of shear rates. Fig. 4 shows the flow curves for OCNF in ChCl–U at different temperatures. The flow curves of the OCNF–DES mixtures with ChCl–Gly and betaine–Gly are shown in Fig. S8 and S9† at different temperatures. In all cases, the viscosity decreases with increasing shear rate. This ‘shear-thinning’ is a favourable gel property for many applications as it makes the material easy to apply but it will then hold its structure. This viscosity decrease for the mixture with betaine–Gly is significantly less (not even one order of magnitude), than for ChCl–U or ChCl–Gly mixtures, both of which decrease by two orders of magnitude across the measured shear range.

**Fig. 5:**
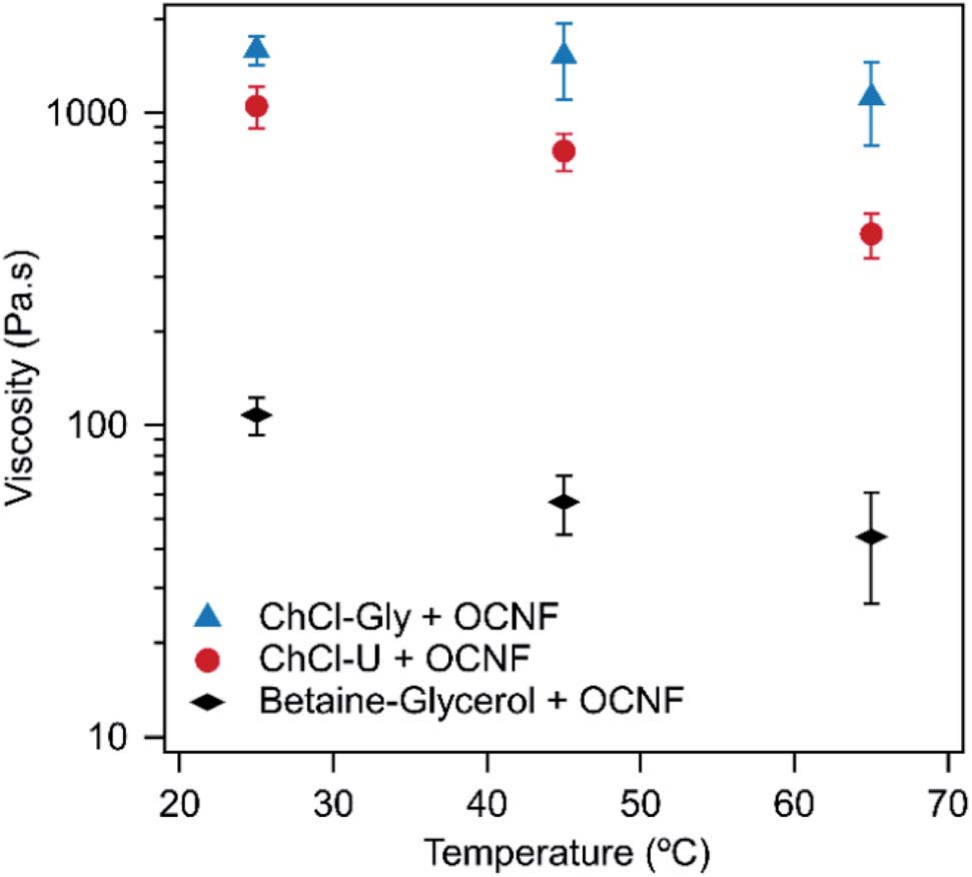
Viscosity of 1.5 wt% OCNF in ChCl–Gly, ChCl–U or betaine–Gly at a shear rate of 0.1 s^−1^ at different temperatures. Error bars are based on the standard deviation of duplicate measurements.

For all three DESs, the viscosity decreases with increasing temperature, although this effect is weak in mixtures with ChCl–Gly. It is possible that large variations between measurements are masking the effect of temperature. These large variations occurred because flow sweeps were difficult to control as even at relatively low shear rates (1 s^−1^), the samples ‘climbed out’ from beneath the plate, a phenomenon which is known for viscoelastic fluids, where configurational entropy is an important contribution to elasticity, *e.g.* polymer melts.^51^ The viscosity of the three pure DESs are known to decrease with temperature, as discussed elsewhere.^52–54^ For example, ChCl–Gly decreases from 1.4 Pa s at 20 °C to 0.069 Pa s at 60 °C while ChCl–U goes from 1 Pa s to 0.054 Pa s across the same temperature range.^52,53^Fig. 5 shows the viscosity of the OCNF:DES mixtures at a single shear rate. Across all temperatures, ChCl–Gly had the highest viscosity, followed by ChCl–U, with betaine–Gly having a much lower viscosity, consistent with it being predominantly a liquid rather than a gel, as discussed above.

As these are soft physical gels rather than chemically cross-linked gels, mechanical measurements such as tensile and compression tests are not appropriate.

The DES–OCNF gels showed distinctive and peculiar differences when compared with the expected behaviour of aqueous OCNF gels. For example, edge failure was observed at relatively small shear rates (1 s^−1^). This behaviour is normally associated with concentrated polymer dispersions, and is not an expected behaviour of nanofibril dispersions. As mentioned earlier in the text, the temperature dependence of *G*′ also suggests conformational entropy as a relevant elastic process, which is also associated with polymeric dispersions.^48^ Thus, one possible explanation is that these OCNF dispersions are behaving somewhat like polymer solutions.

Under shear, polymer chains are known to stretch and disentangle, and as a way to minimise entropy, will migrate to regions of lower shear, exerting a stress normal to the surface of the geometry in the process of migrating.^55^ This provides a possible explanation for the peculiar behaviour observed for the DES–OCNF gels. As a general rule of thumb, cellulose derivatives with free –OH groups are not soluble in water, due to formation of a strong and extensive network of inter-cellulose hydrogen bonds,^56^ thus aqueous OCNF gels behave as particle gels. However, DES formation relies on species capable of extensive hydrogen bonding. Therefore, it is possible that the DESs are competing with the inter-cellulose hydrogen bonding and partly solubilising the OCNF fibrils, to a degree where patches of entanglements associated with the fibril network are present, which was observed in cases of partial solubilisation of cellulose.^56^

Furthermore, OCNF is composed of mixed crystalline or amorphous regions and previous work has shown that DESs can reduce the crystallinity of cellulose.^21^ Previous papers have also shown that ChCl–U and ChCl–Gly can solubilise small concentrations of cellulose, although this usually required extensive heating.^57^ Alternatively, it is also possible that the choline cation is reacting with the carboxyl portions of the OCNF, such ester formation is known in the literature.^58^ This would modify the cellulose fibrils, potentially leading to zwitterionic fibrils, and possibly disrupting the fibril surface structure while also modifying fibril–fibril interactions. For example, some fibrils could retain the negative carboxylate group while others will have the positive ammonium group, leading to strong fibril–fibril association. Water is also released during the condensation reaction, which may modify the solubility of the Na^+^ ions in these solutions. Additionally it is known that choline undergoes degradation *via* a polymerisation route, forming poly(ethylene glycol) (PEG) species with time.^59^ The presence of PEG oligomers may also alter the rheological response of the choline chloride containing gels compared to those containing the betaine species.

While the above hypothesis fit with the data, it is impossible to be certain of structural features based just on the rheological data and there may be other explanations. Further work is required to better investigate the structure of these systems, including identifying any reactions that might have occurred and any changes to the crystalinity or flexibility of the cellulose fibrils. The low concentration of OCNF particles in the gels (1 wt%) prevents direct measurement of cellulose crystallinity in these materials using powder XRD techniques on lab-based sources, so synchrotron experiments would be required. Similarly, in FTIR, the OCNF signals were obscured by signals from the much more concentrated DESs.

### SAXS

**Fig. 6:**
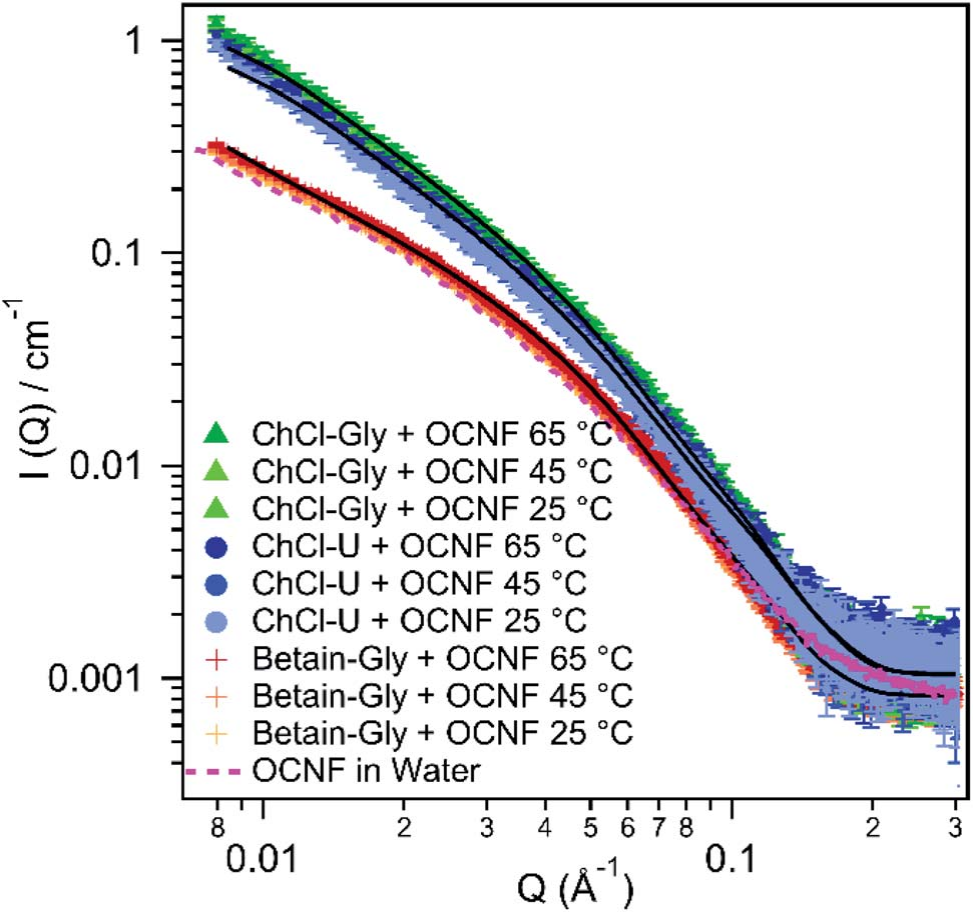
SAXS patterns of 1.5 wt% OCNF in either ChCl–U, ChCl–Gly or betaine–Gly at 25, 45, or 65 °C. 1.5 wt% OCNF in water at 25 °C is shown with a pink dashed line. Fits are shown with solid black lines.

Small angle X-ray scattering was performed to get a better understanding of the interactions and structure of these OCNF–DES mixtures at the nanoscale. Fig. 6 shows the scattering patterns of all three mixtures at 25 °C, 45 °C, or 65 °C. Fitting parameters are given in the ESI.†

None of the mixtures displayed changes in their small angle scattering pattern with changes in temperature. Therefore, the rheological and conductivity changes observed with increasing temperature (and discussed above and below) are not a result of structural changes at this length scale.

Previous research on OCNF dispersions in water, fitted small angle X-ray scattering data using an elliptical cylinder model representing the fibril, with a minor radius of 14 ± 1 Å and a major radius of 51 ± 1 Å.^33^ The pink dashed line in Fig. 6 shows the scattering pattern for 1.5 wt% OCNF in water. It is almost identical to the scattering pattern of OCNF in betaine–Gly and in both cases the data can be fit with an elliptical cylinder model. The data for 1.5 wt% OCNF in betaine–Gly can be fit with a minor radius of 16 ± 2 Å and a major radius of 53 ± 6 Å which is the same within the error of the fit for OCNF in water.

In contrast, the data for OCNF in combination with ChCl–U or ChCl–Gly can be better fit with a flexible elliptical cylinder model instead of a rigid one. This same flexible cylinder model has been used previously by our group to model OCNF in water in the presence of salt.^33^ This is because salt ions interrupt fibril–fibril repulsion from the negatively charged carboxylate groups and allow closer associations. This manifests as interstices where fibrils cross, which in SAXS are indistinguishable from flexible rods.

For OCNF in ChCl–Gly and ChCl–U, the minor and major radii were much the same as the sample in betaine–Gly (see Table 1), but with a flexible Kuhn length. This Kuhn length could be varied from 100–200 Å without significantly changing the radii or the quality of the fit. This Kuhn length is similar to that observed for OCNF in water with the addition of salt.^33^ It is also the same as that reported for zwitterionic cellulose nanofibrils,^60^ and so would be consistent with either the DES ions allowing closer association of OCNF fibrils, or with choline reacting with OCNF to create zwitterionic fibrils which can associate more closely than purely anionic fibrils. If the data for ChCl–Gly and ChCl–U are fit with a rigid elliptical cylinder model then the major radii significantly increases to 80 ± 10 Å, however the Chi2 values suggest this is not a suitable fit (see ESI†).

**Table 1:** Fitting parameters of 1.5 wt% OCNF in H_2_O, ChCl–U, ChCl–Gly, or betaine–Gly at 25 °C

	Model	Minor radius [Å]	Major radius [Å]	Kuhn length [Å]
OCNF + H_2_O	Elliptical cylinder	14 ± 1	51 ± 1	N/A
OCNF + H_2_O + 0.1 M NaCl^33^	Flexible elliptical cylinder	14 ± 1	51 ± 1	240 ± 20
OCNF + ChCl–U	Flexible elliptical cylinder	16 ± 2	48 ± 6	100–200
OCNF + ChCl–Gly	Flexible elliptical cylinder	16 ± 2	51 ± 6	100–200
OCNF + betaine–Gly	Elliptical cylinder	16 ± 2	53 ± 6	N/A

The fact that the scattering pattern of OCNF in betaine–Gly is almost indistinguishable from that of OCNF in water suggests that the betaine–Gly DES is not having the same effect on the fibrils as the choline chloride based ones. This could mean that the zwitterionic betaine molecule is unable to negate the repulsive forces between the OCNF fibrils. Alternatively, it could indicate that the choline cation is indeed reacting with the fibrils, something that betaine is unable to do. The presence of separate charges (*i.e.* choline chloride compared to betaine) also appears to have a significant influence on the conductivity of these OCNF:DES mixtures.

### Conductivity

**Fig. 7:**
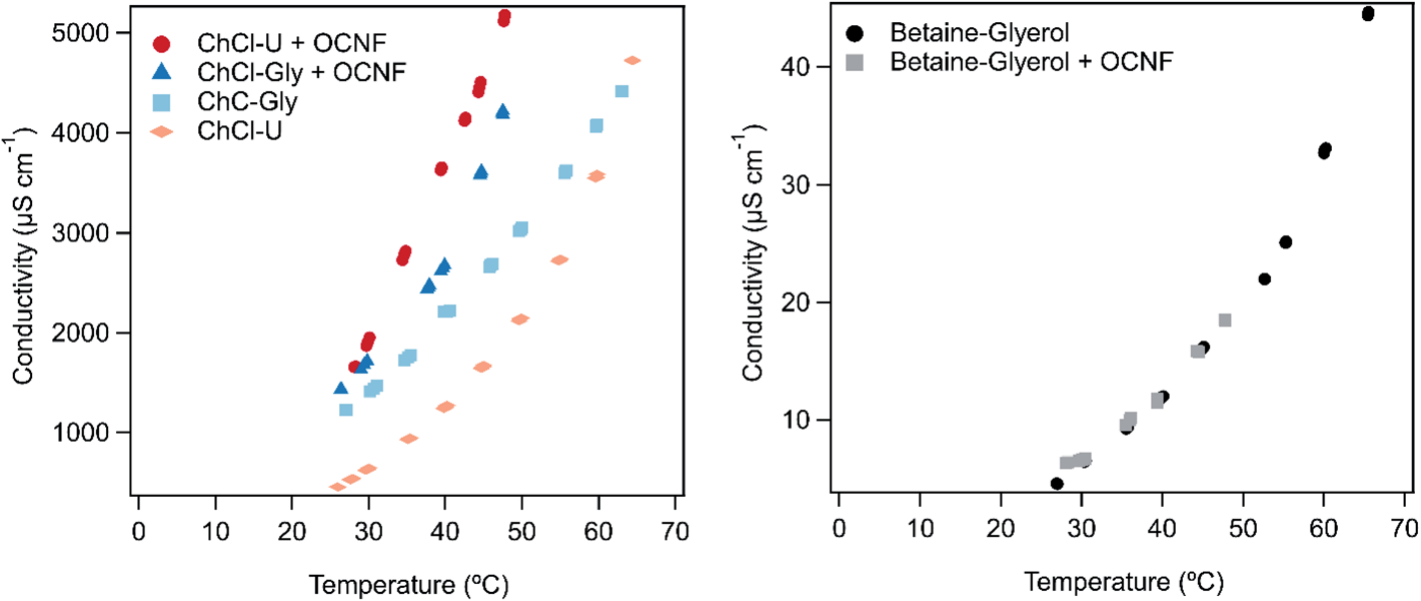
Conductivity of ChCl–U and ChCl–Gly (left) and betaine–Gly (right) with and without 1.5 wt% OCNF across a range of temperatures. Measurements at each temperature were performed in triplicate on each sample, and all are plotted on the above graph, demonstrating the consistency of the results.

There is a lot of interest in DESs for electronics and batteries.^1,2,15,25^ Their ionic nature and non-volatility make them ideal candidates for these applications. The mixtures presented in this work offer a unique combination of non-volatility (so they will not evaporate if left exposed to the air), and tuneable gel-like properties (so they can be applied as creams or gels, without the risk of dripping that would come from liquid samples). Not only that, but these gels are conductive. Fig. 7 shows the conductivity of the DESs on their own and in combination with OCNF across a range of temperatures. Errors reported here are based on the reported instrumental error of 0.5%.

There is some inconsistency in the literature around the conductivity of different deep eutectic solvents. It is possible that this arises from the presence of small amounts of water which could greatly increase conductivity due to decreased viscosity and increased ion movement, or slight molar imbalances in the DESs themselves, or inaccurate temperature readings. The conductivity of ChCl–U at room temperature has been reported as 1.8 mS cm^−1^,^61^ and ∼0.4 mS cm^−1^,^62^ while a value of 0.199 mS cm^−1^ was reported at 40 °C.^63^ We found the conductivity of ChCl–U at room temperature to be 0.4623 ± 0.002 mS cm^−1^ which is within the range reported in the literature. Similarly, the conductivity of ChCl–Gly at room temperature has been reported as 1.4 mS cm^−1^,^61^ ∼1 mS cm^−1^ and 1.05 mS cm^−1^ at 20 °C.^64^ We found the conductivity of ChCl–Gly to be 1.2273 ± 0.002 mS cm ^−1^, again within the range reported in the literature. We were unable to find references for the conductivity of betaine–Gly in the literature, but measured it at just 0.00461 ± 0.00003 mS cm^−1^ at room temperature which is consistent with the lack of free small ions in this DES.

As shown in Fig. 7, the conductivity of all samples increased with increasing temperature. This is most likely due to a decrease in viscosity (as demonstrated in the above rheological measurements).^65,66^

The conductivity of both ChCl–U and ChCl–Gly increased upon addition of 1.5 wt% OCNF (up to 1.661 ± 0.008 mS cm^−1^ and 1.4317 ± 0.007 mS cm^−1^ respectively at room temperature), although the conductivity of ChCl–U increased to a greater degree. Meanwhile, the conductivity of betaine–Gly did not appear to change at all upon addition of OCNF. An increase in conductivity upon addition of OCNF is against expectations because the increase in viscosity and gel-like nature of the DESs with OCNF should decrease the ability of ions to move through the sample. Therefore, some new process is taking place besides the changes in viscosity. The OCNF used in this work is a sodium salt. The sodium ions present on the OCNF could increase the conductivity of samples, however, the fact that the conductivity of the betaine–Gly mixture did not change suggests that this is not the cause.

The nanostructure of DESs, especially ChCl–U and ChCl–Gly has been well studied. Investigations involving addition of water,^18,67^ and computer simulations of polymer solvation,^68^ have demonstrated unexpected disruptions and instabilities of the DES network. In addition, the hydrogen bonding capacity of cellulose is well known. Therefore, it is quite possible that both glycerol and urea will preferentially interact with OCNF, releasing Na^+^ from the fibrils and Cl^−^ from the DES, thus creating more charge carriers and increasing conductivity. The fact that no increase in conductivity is seen for betaine–Gly + OCNF suggests that the release of Cl^−^ is much more significant than the effects of Na^+^.

Additionally, as discussed above, if the choline cation is reacting with the OCNF fibrils, this would release Na^+^ from the fibril surfaces, and liberate water during the reaction but would not occur in the betaine–Gly, thus further contributing to the higher conductivity of choline chloride based DESs with OCNF.

As shown in Fig. 7, the conductivity of ChCl–Gly changed less than ChCl–U on addition of OCNF. This is probably a viscosity effect. ChCl–U + OCNF has a much lower viscosity than ChCl–Gly + OCNF (Fig. 5), which would in turn allow it to have greater conductivity with the same number of free ions.

The conductivity of these gels is much greater than that of similar systems presented in the literature. Other work using starch and ChCl–U to make an elastic solid found a conductivity of only 0.218 μS cm^−1^.^69^ Similarly, gels formed using 1,3:2,4-dibenzylidene-d-sorbitol (DBS) had conductivities of ∼0.2 mS cm^−1^ when made with ChCl–U and 0.9 mS cm^−1^ when made with ChCl–Gly.^62^ Higher conductivities have been achieved in gels made from ChCl–ethylene glycol with chemically cross-linked methacrylate polymers (5.7 mS cm^−1^)^25^ or using gelatin (2.5 mS cm^−1^).^3^ However, chemical crosslinking of methacrylates is a far more expensive and environmentally damaging method of gel formation than using cellulose, and the animal source of gelatin makes it less than ideal due to ethical and religious concerns. It would be interesting to measure the conductivity of gels made from OCNF with ChCl–ethylene glycol as this DES already has a higher conductivity than any of the three DESs explored in this research. Such a mixture could have comparable conductivities to the above-mentioned gels made with methacrylate and gelatin.

## Conclusion

This research demonstrates a novel two-component eutectogel made from DESs and nanocellulose fibrils. These gels are shear thinning which makes them ideal for applications including pumping and spreading. There was no evidence of gel breakdown or syneresis during any of the characterisation steps, demonstrating their stability. Furthermore, the non-volatility of the DESs means that these gels could be used in the open air without risk of evaporation, including at elevated temperature. These DESs may also allow applications at temperatures below freezing as they can undergo vitrification. All of the components tested in these gels have so far been found to be non-toxic and non-hazardous and could therefore be used for pharmaceutical gels and applications, for example as drug delivery vehicles on the skin.

## Conflicts of interest

The authors declare no conflict of interest.

## Supplementary Material

NA-003-D0NA00976H-s001

## References

[cit1] Lloyd D., Vainikka T., Kontturi K. (2013). Electrochim. Acta.

[cit2] Joos B., Vranken T., Marchal W., Safari M., Van Bael M. K., Hardy A. T. (2018). Chem. Mater..

[cit3] Qin H., Owyeung R. E., Sonkusale S. R., Panzer M. J. (2019). J. Mater. Chem. C.

[cit4] Lee H.-R., Woo J., Han S. H., Lim S.-M., Lim S., Kang Y.-W., Song W. J., Park J.-M., Chung T. D., Joo Y.-C., Sun J.-Y. (2019). Adv. Funct. Mater..

[cit5] Yang B., Yuan W. (2019). ACS Appl. Mater. Interfaces.

[cit6] Trivedi T. J., Bhattacharjya D., Yu J.-S., Kumar A. (2015). ChemSusChem.

[cit7] Visentin A. F., Panzer M. J. (2012). ACS Appl. Mater. Interfaces.

[cit8] D'Angelo A. J., Grimes J. J., Panzer M. J. (2015). J. Phys. Chem. B.

[cit9] Zhu M., He S., Dai Y., Han J., Gan L., Liu J., Long M. (2018). ACS Sustainable Chem. Eng..

[cit10] Kim Y. M., Moon H. C. (2020). Adv. Funct. Mater..

[cit11] Takada A., Kadokawa J.-i. (2015). Biomolecules.

[cit12] Thuy Pham T. P., Cho C.-W., Yun Y.-S. (2010). Water Res..

[cit13] Abbott A. P., Capper G., Davies D. L., Rasheed R. K., Tambyrajah V. (2003). Chem. Commun..

[cit14] Paiva A., Craveiro R., Aroso I., Martins M., Reis R. L., Duarte A. R. C. (2014). ACS Sustainable Chem. Eng..

[cit15] Zhang Q., De Oliveira Vigier K., Royer S., Jerome F. (2012). Chem. Soc. Rev..

[cit16] Wen Q., Chen J.-X., Tang Y.-L., Wang J., Yang Z. (2015). Chemosphere.

[cit17] Radošević K., Cvjetko Bubalo M., Gaurina Srček V., Grgas D., Landeka Dragičević T., Radojčić Redovniković I. (2015). Ecotoxicol. Environ. Saf..

[cit18] Hammond O. S., Bowron D. T., Edler K. J. (2017). Angew. Chem., Int. Ed..

[cit19] Hammond O. S., Bowron D. T., Jackson A. J., Arnold T., Sanchez-Fernandez A., Tsapatsaris N., Garcia Sakai V., Edler K. J. (2017). J. Phys. Chem. B.

[cit20] Zdanowicz M., Staciwa P., Jędrzejewski R., Spychaj T. (2019). Polymers.

[cit21] Wang S., Peng X., Zhong L., Jing S., Cao X., Lu F., Sun R. (2015). Carbohydr. Polym..

[cit22] Zdanowicz M., Spychaj T. (2011). Polimery.

[cit23] Mukesh C., Gupta R., Srivastava D. N., Nataraj S. K., Prasad K. (2016). RSC Adv..

[cit24] Mukesh C., Upadhyay K. K., Devkar R. V., Chudasama N. A., Raol G. G., Prasad K. (2016). Macromol. Chem. Phys..

[cit25] Qin H., Panzer M. J. (2017). ChemElectroChem.

[cit26] Marullo S., Meli A., Giannici F., D'Anna F. (2018). ACS Sustainable Chem. Eng..

[cit27] Isogai A., Saito T., Fukuzumi H. (2011). Nanoscale.

[cit28] Saito T., Nishiyama Y., Putaux J.-L., Vignon M., Isogai A. (2006). Biomacromolecules.

[cit29] Fujisawa S., Okita Y., Fukuzumi H., Saito T., Isogai A. (2011). Carbohydr. Polym..

[cit30] Wu B., Geng B., Chen Y., Liu H., Li G., Wu Q. (2017). Front. Chem. Sci. Eng..

[cit31] Hastuti N., Kanomata K., Kitaoka T. (2019). IOP Conf. Ser. Earth Environ. Sci..

[cit32] Calabrese V., Muñoz-García J. C., Schmitt J., da Silva M. A., Scott J. L., Angulo J., Khimyak Y. Z., Edler K. J. (2019). J. Colloid Interface Sci..

[cit33] Schmitt J., Calabrese V., da Silva M. A., Lindhoud S., Alfredsson V., Scott J. L., Edler K. J. (2018). Phys. Chem. Chem. Phys..

[cit34] Crawford R. J., Edler K. J., Lindhoud S., Scott J. L., Unali G. (2012). Green Chem..

[cit35] da Silva M. A., Calabrese V., Schmitt J., Celebi D., Scott J. L., Edler K. J. (2018). Soft Matter.

[cit36] Fukuzumi H., Tanaka R., Saito T., Isogai A. (2014). Cellulose.

[cit37] Lasseuguette E., Roux D., Nishiyama Y. (2008). Cellulose.

[cit38] Goi Y., Fujisawa S., Saito T., Yamane K., Kuroda K., Isogai A. (2019). Langmuir.

[cit39] Mendoza L., Batchelor W., Tabor R. F., Garnier G. (2018). J. Colloid Interface Sci..

[cit40] Lin N., Dufresne A. (2014). Eur. Polym. J..

[cit41] Celebi D., Guy R. H., Edler K. J., Scott J. L. (2016). Int. J. Pharm..

[cit42] Zhu L., Kumar V., Banker G. S. (2004). AAPS PharmSciTech.

[cit43] Ahmadi R., Hemmateenejad B., Safavi A., Shojaeifard Z., Mohabbati M., Firuzi O. (2018). Chemosphere.

[cit44] Hayyan M., Looi C. Y., Hayyan A., Wong W. F., Hashim M. A. (2015). PloS One.

[cit45] Macário I. P. E., Oliveira H., Menezes A. C., Ventura S. P. M., Pereira J. L., Gonçalves A. M. M., Coutinho J. A. P., Gonçalves F. J. M. (2019). Sci. Rep..

[cit46] Courtenay J. C., Johns M. A., Galembeck F., Deneke C., Lanzoni E. M., Costa C. A., Scott J. L., Sharma R. I. (2017). Cellulose.

[cit47] Verdanova M., Pytlik R., Kalbacova M. H. (2014). Biopreserv. Biobanking.

[cit48] Kavanagh G. M., Ross-Murphy S. B. (1998). Prog. Polym. Sci..

[cit49] Lieleg O., Claessens M. M. A. E., Luan Y., Bausch A. R. (2008). Phys. Rev. Lett..

[cit50] Pandey A., Pandey S. (2014). J. Phys. Chem. B.

[cit51] Schramm G. , A Practical Approach to Rheology and Rheometry, Gebrueder HAAKE GmbH, Federal Republic of Germany, 2000

[cit52] Yadav A., Pandey S. (2014). J. Chem. Eng. Data.

[cit53] Yadav A., Trivedi S., Rai R., Pandey S. (2014). Fluid Phase Equilib..

[cit54] Kučan K. Z., Perković M., Cmrk K., Načinović D., Rogošić M. (2018). ChemistrySelect.

[cit55] Sunthar P. , in Rheology of Complex Fluids, ed. J. M. Krishnan, A. P. Deshpande and P. B. S. Kumar, Springer New York, New York, NY, 2010, pp. 171–191

[cit56] Klemm D., Heublein B., Fink H.-P., Bohn A. (2005). Angew. Chem., Int. Ed..

[cit57] Sharma M., Mukesh C., Mondal D., Prasad K. (2013). RSC Adv..

[cit58] Rodriguez Rodriguez N., van den Bruinhorst A., Kollau L. J. B. M., Kroon M. C., Binnemans K. (2019). ACS Sustainable Chem. Eng..

[cit59] Kirk R. E. , Othmer D. F., Kroschwitz J. I. and Howe-Grant M., Kirk-Othmer encyclopedia of chemical technology, John Wiley and Sons, 2000

[cit60] Calabrese V., da Silva M. A., Schmitt J., Muñoz-Garcia J. C., Gabrielli V., Scott J. L., Angulo J., Khimyak Y. Z., Edler K. J. (2018). Soft Matter.

[cit61] Selvanathan V., Azzahari A. D., Halim A. A. A., Yahya R. (2017). Carbohydr. Polym..

[cit62] Ruiz-Olles J., Slavik P., Whitelaw N. K., Smith D. K. (2019). Angew. Chem., Int. Ed..

[cit63] Abbott A. P., Capper G., Gray S. (2006). ChemPhysChem.

[cit64] Abbott A. P., Harris R. C., Ryder K. S. (2007). J. Phys. Chem. B.

[cit65] Al-Murshedi A. Y. M., Alesary H. F., Al-Hadrawi R. (2019). J. Phys. Conf..

[cit66] García G., Aparicio S., Ullah R., Atilhan M. (2015). Energy Fuels.

[cit67] Weng L., Toner M. (2018). Phys. Chem. Chem. Phys..

[cit68] Stefanovic R., Webber G. B., Page A. J. (2019). J. Mol. Liq..

[cit69] Abbott A. P., Ballantyne A. D., Conde J. P., Ryder K. S., Wise W. R. (2012). Green Chem..

