# Supplementary material for: Non-volatile conductive gels made from deep eutectic solvents and oxidised cellulose nanofibrils
Source: Nanoscale Adv. 2021 Mar 2;3(8):2252–60. doi: 10.1039/d0na00976h (PMC9419570; doi:10.1039/d0na00976h)
Supplement: NA-003-D0NA00976H-s001 [file NA-003-D0NA00976H-s001.pdf]

Electronic Supplementary Material (ESI) for Nanoscale Advances.
This journal is © The Royal Society of Chemistry 2021

Non-Volatile Conductive Gels Made From Deep Eutectic Solvents and Oxidised Cellulose Nanofibrils

Saffron J. Bryant,[a,c*] *Marcelo A. da Silva,*[a] *Kazi M. Zakir Hossain,*[a] *Vincenzo Calabrese,*[a] *Janet L. Scott,*[a,b] *Karen J. Edler*[a *]

[a] Department of Chemistry, University of Bath, Claverton Down, Bath, BA2 7AY, United Kingdom

[b] Centre for Sustainable Chemical Technologies, University of Bath, Claverton Down, Bath, BA2 7AY, United Kingdom

[c] School of Science, College of Science, Engineering and Health, RMIT University, Melbourne VIC 3001, Australia

saffron.bryant@rmit.edu.au, k.edler@bath.ac.uk

SAXS

The following table show the best-fit parameters for DES and OCNF combinations. All fitting was done in SASView (Version 4.2.1, see http://www.sasview.org/ for more information). The models utilized were not modified from their implementation in SASView 4.2.1:

Elliptical Cylinder – This fit is catalogued under "Cylinder Functions".[1]

Flexible Cylinder Elliptical – This fit is catalogued under "Cylinder Functions".[2, 3]

Table S1 shows the fitting parameters for both the elliptical cylinder model and the flexible elliptical cylinder model for all DES/OCNF combinations for comparison. However, based on the qualities of the fits, the chi2 values and the obtained parameters, the flexible elliptical cylinder model is more appropriate for mixtures of OCNF with ChCl-U and ChCl-Gly, while the elliptical cylinder model is better for OCNF with Betaine-Gly,

Table S1. Fitting parameters for 1.5 wt% OCNF in deep eutectic solvents. (In all cases the length was fixed at 1000 Å).

	Model	Scale	Bkg	Minor Radius	Major Radius	Kuhn Length	Chi2
ChCl-Gly + OCNF 25 °C	Elliptical cylinder	0.00076	0.001	16±2	82±10		1.4326
	Flexible elliptical cylinder	0.00022	0.001	16±2	51±6	100-200	1.1385
ChCl-Gly + OCNF 45 °C	Elliptical cylinder	0.00078	0.001	16±2	82±10		1.4299
	Flexible elliptical cylinder	0.00023	0.001	16±2	50±6	100-200	1.2157
ChCl-Gly + OCNF 65 °C	Elliptical cylinder	0.00085	0.001	16±2	88±11		2.6799
	Flexible elliptical cylinder	0.00023	0.001	15±2	51±7	100-200	1.4339
ChCl-U + OCNF 25 °C	Elliptical cylinder	0.0011	0.001	16±2	78±10		1.4925
	Flexible elliptical cylinder	0.00035	0.001	16±2	48±6	100-200	1.177
ChCl-U + OCNF 45 °C	Elliptical cylinder	0.00118	0.001	15±2	73.5±10		1.4527
	Flexible elliptical cylinder	0.00037	0.001	15±2	45±6	100-200	1.1286
ChCl-U + OCNF 65 °C	Elliptical cylinder	0.0013	0.001	15±2	87±12		1.5776
	Flexible elliptical cylinder	0.00035	0.001	14±2	49±7	100-200	1.3546
Betain-Glycerol + OCNF 25 °C	Elliptical cylinder	0.00062	0.001	16±2	53±7		1.9463
	Flexible elliptical cylinder	0.00019	0.001	16±2	51±6	500-600	1.8135

Betain-Glycerol + OCNF 45 °C	Elliptical cylinder	0.00065	0.0007	15±2	51±7		3.4479
	Flexible elliptical cylinder	0.00020	0.0007	16±2	51±6	500-600	3.4907
Betain-Glycerol + OCNF 65 °C	Elliptical cylinder	0.00068	0.0008	16±2	5451±7		2.9384
	Flexible elliptical cylinder	0.00021	0.0008	16±2	51±6	500-600	2.7928

Rheology

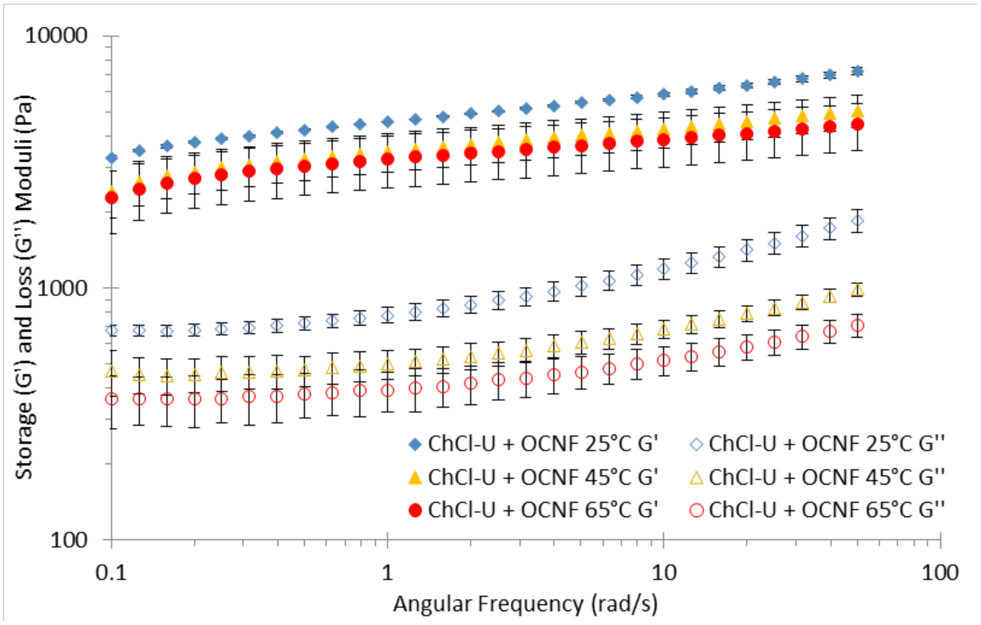

Figure S1. Frequency sweep curves of 1.5 wt% OCNF in ChCl-U at 25, 45 and 65 °C.

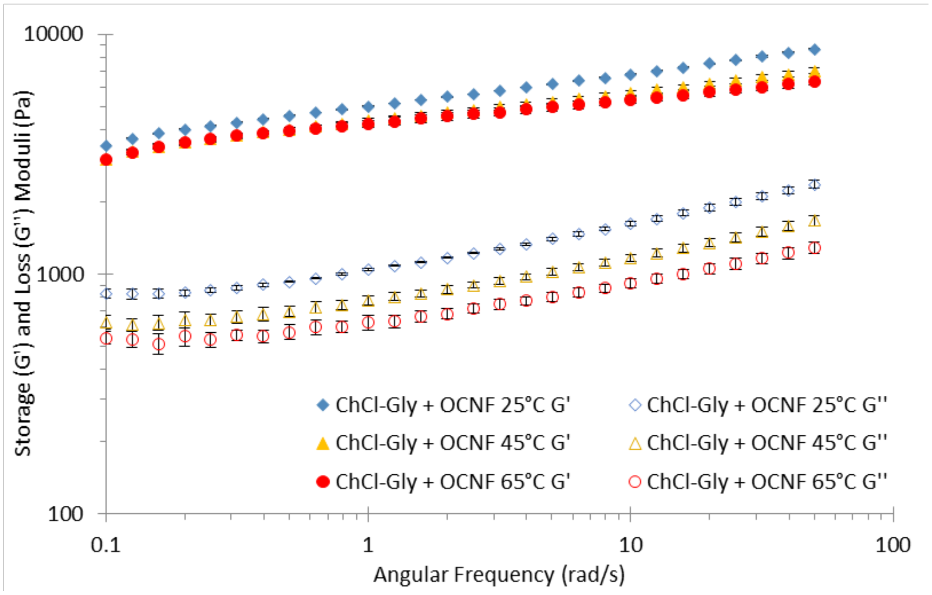

Figure S2. Frequency sweep curves of 1.5 wt% OCNF in ChCl-Gly at 25, 45 and 65 °C.

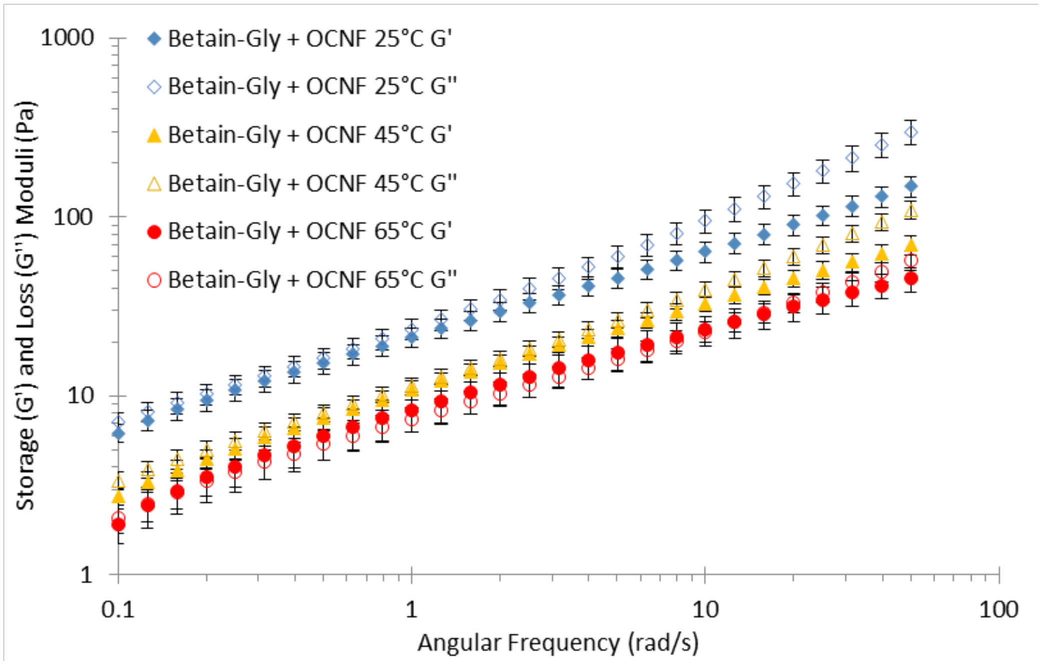

Figure S3. Frequency sweep curves of 1.5 wt% OCNF in Betaine-Gly at 25, 45 and 65 °C.

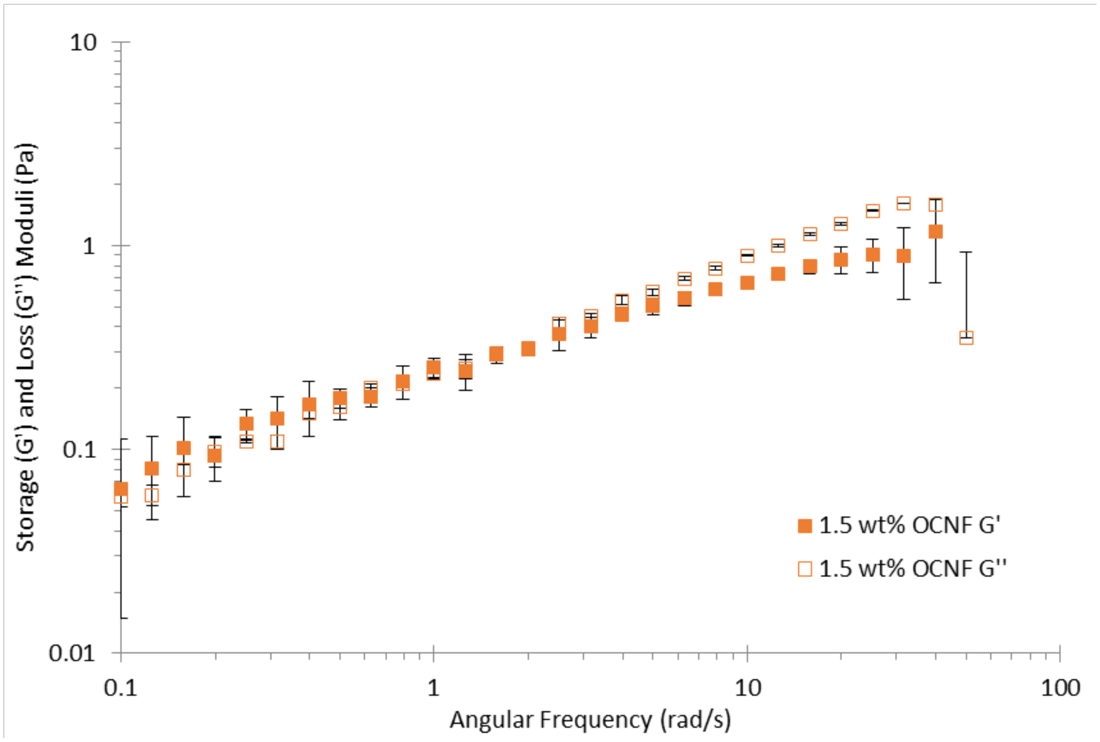

Figure S4. Frequency sweep curves of 1.5 wt% OCNF in water.

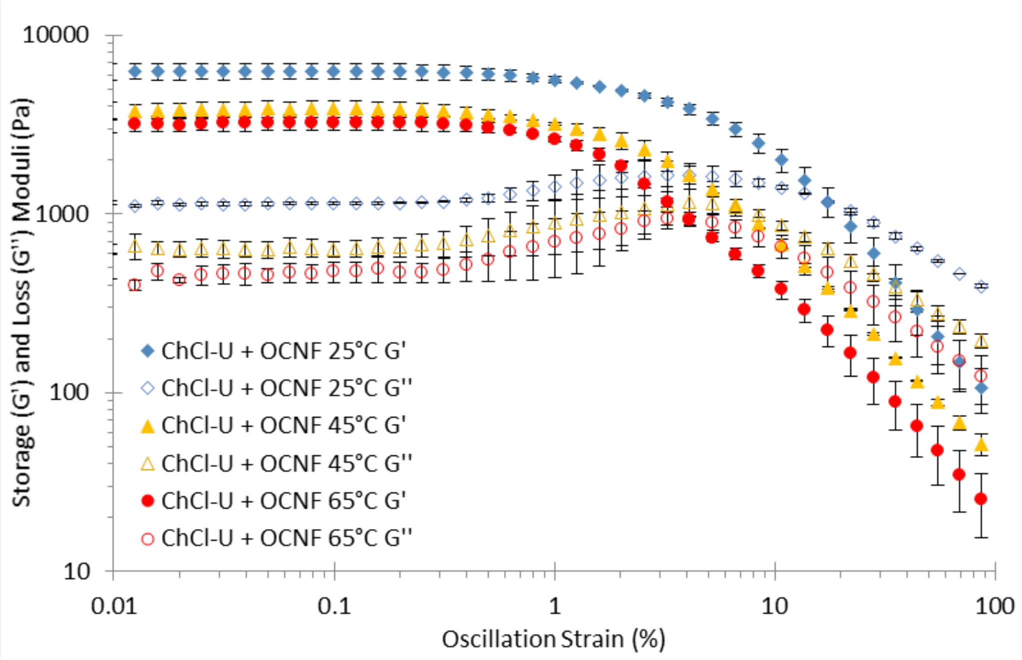

Figure S5. Amplitude sweep curves of 1.5 wt% OCNF in ChCl-U at 25, 45, and 65 °C.

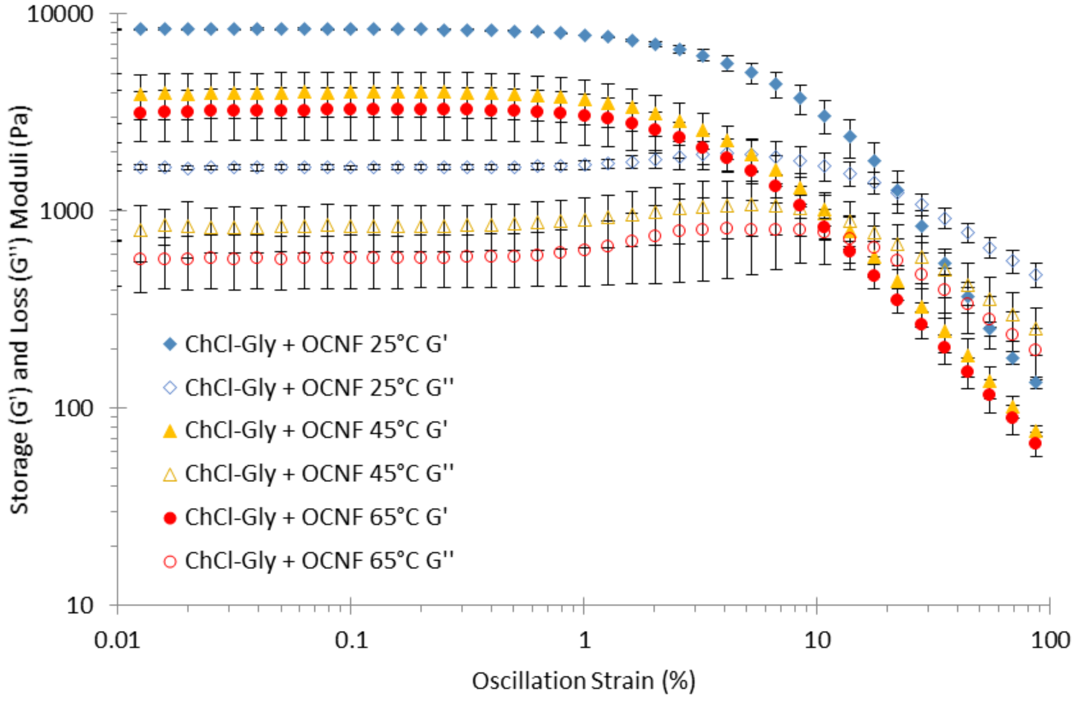

Figure S6. Amplitude sweep curves of 1.5 wt% OCNF in ChCl-Gly at 25, 45, and 65 °C.

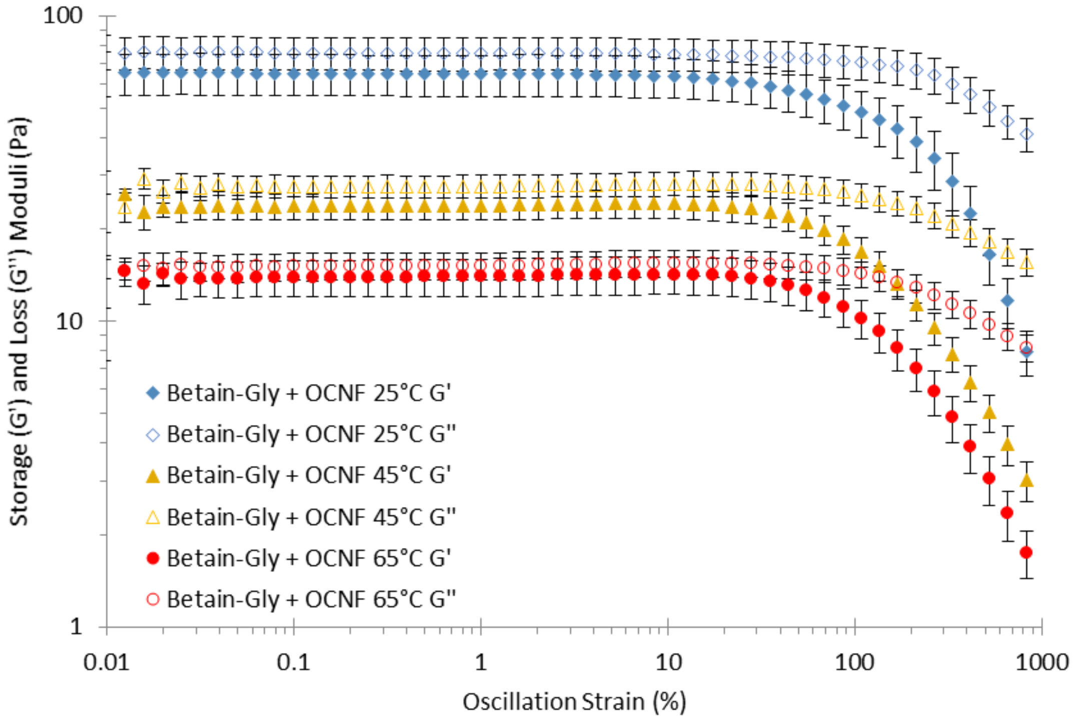

Figure S7. Amplitude sweep curves of 1.5 wt% OCNF in Betain-Gly at 25, 45, and 65 °C.

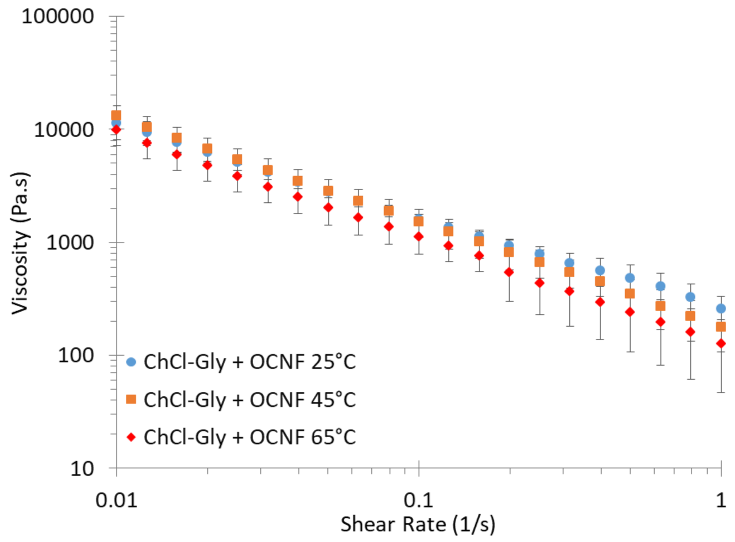

Figure S8. Flow curves of 1.5 wt% OCNF in ChCl-Gly at 25, 45, or 65 °C.

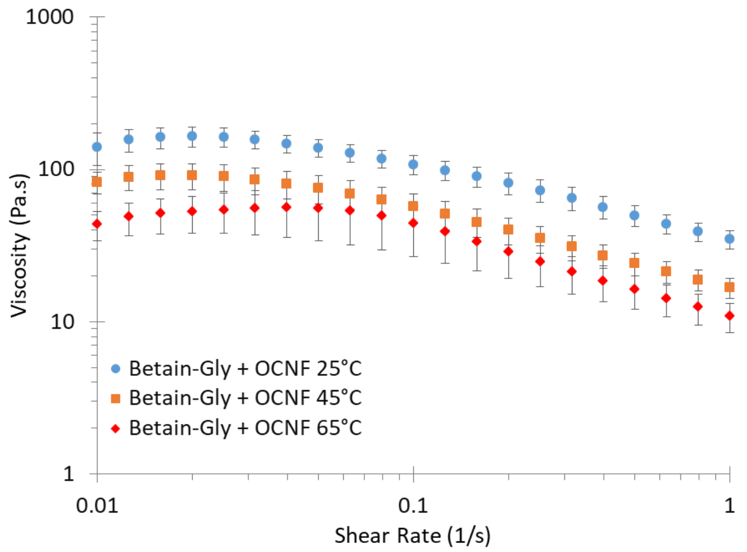

Figure S9. Flow curves of 1.5 wt% OCNF in Betaine-Gly at 25, 45, or 65 °C.

References

1. L. A. Feigin and D. I. Svergun, *Structure Analysis by Small-Angle X-Ray and Neutron Scattering*, Springer US, 1987.
2. J. S. Pedersen and P. Schurtenberger, *Macromolecules*, 1996, **29**, 7602-7612.
3. W.-R. Chen, P. D. Butler and L. J. Magid, *Langmuir*, 2006, **22**, 6539-6548.
